# Potential drug–drug interactions associated with adverse clinical outcomes and abnormal laboratory findings in patients with malaria

**DOI:** 10.1186/s12936-020-03392-5

**Published:** 2020-08-31

**Authors:** Sidra Noor, Mohammad Ismail, Faiza Khadim

**Affiliations:** Department of Pharmacy, University of Peshawar, Khyber Pakhtunkhwa, Pakistan

**Keywords:** Patient safety, Malaria, Clinical relevance, Potential drug–drug interactions, Polypharmacy

## Abstract

**Background:**

Hospitalized patients with malaria often present with comorbidities or associated complications for which a variety of drugs are prescribed. Multiple drug therapy often leads to drug–drug interactions (DDIs). Therefore, the current study investigated the prevalence, levels, risk factors, clinical relevance, and monitoring parameters/management guidelines of potential DDIs (pDDIs) among inpatients with malaria.

**Methods:**

A retrospective cohort study was carried out at two tertiary care hospitals. A total of 398 patients’ profiles were evaluated for pDDIs using the Micromedex Drug-Reax^®^. Odds ratios were calculated to identify the strength of association between presence of DDIs and potential risk factors via logistic regression analysis. Further, the clinical relevance of frequent pDDIs was investigated.

**Results:**

Of 398 patients, pDDIs were observed in 37.2% patients, while major-pDDIs in 19.3% patients. A total of 325 interactions were found, of which 45.5% were of major- and 34.5% moderate-severity. Patients with the most common pDDIs were found with signs/symptoms and abnormalities in laboratory findings representing nephrotoxicity, hepatotoxicity, QT interval prolongation, and reduced therapeutic efficacy. The following drug pairs reported the highest frequency of adverse events associated with the interactions; calcium containing products-ceftriaxone, isoniazid–rifampin, pyrazinamide–rifampin, isoniazid–acetaminophen, and ciprofloxacin–metronidazole. The adverse events were more common in patients prescribed with the higher doses of interacting drugs. Multivariate regression analysis showed statistically significant association of pDDIs with 5–6 prescribed medicines (p = 0.01), > 6 prescribed medicines (p < 0.001), > 5 days of hospital stay (p = 0.03), and diabetes mellitus (p = 0.04).

**Conclusions:**

PDDIs are commonly observed in patients with malaria. Healthcare professional’s knowledge about the most common pDDIs could help in preventing pDDIs and their associated negative effects. Pertinent clinical parameters, such as laboratory findings and signs/symptoms need to be checked, particularly in patients with polypharmacy, longer hospital stay, and diabetes mellitus.

## Background

Malaria is one of the infectious diseases that cause burden on the healthcare system. According to the latest World Health Organization (WHO) report released in 2019, malaria accounts for 228 million cases worldwide in 2018 with an estimated number of deaths 405,000 [1]. In Pakistan, approximately 70,5000 cases of malaria have been reported in 2018 [2]. Worldwide, malaria remains one of the causes of death due to infectious diseases [3]. Population group that are more exposed to malaria include male gender, > 14 years of age, and rural population [4].

Hospitalization in malaria occurs due to disease severity, managing the associated symptoms or comorbid illnesses [5]. Anti-malarial drugs, anti-pyretic, and analgesics are usually prescribed to treat hospitalized malaria patients [6]. Besides these medicines, a variety of other medicines are also prescribed so as to manage the comorbid illnesses and associated symptoms [5–7]. Concomitant use of several drugs increased the chance of drug–drug interactions (DDIs)-affecting drug’s pharmacokinetic parameters and pharmacodynamics profile [8, 9]. DDIs may lead to a variety of negative clinical outcomes such as hospitalization, reduced or abolished therapeutic efficacy, prolongation of hospital stay, toxicity, and adverse effects [8–10]. An approximately, 20–30% of adverse effects have been reported as due to DDIs, of which 1–2% are life-threatening and 70% need clinical intervention [11]. Hence, particular consideration of DDIs and their timely management is crucial for the rational use of medicines in patients with malaria.

Potential DDIs (pDDIs) issue has been addressed generally in hospitalized patients [8] as well as in specific diseases such as liver cirrhosis [12], hypertension [13], diabetes mellitus (DM) [14], bone marrow transplant [15], cancer [16], stroke [17], pneumonia [18], urinary tract infections [19], and hepatitis C [20]. Despite, being one of the most prevalent causes of hospitalization in Pakistan during its emerging season [21]. DDIs particularly among inpatients with malaria remains unaddressed. Moreover, in developing countries, literature has been least reported as well as irrational use of medicines is a common issue. Consequently, specific consideration is required to conduct studies evaluating pDDIs and their clinical relevance among hospitalized patients with malaria. Afterward, such studies will improve patients’ safety and help healthcare professionals to manage pDDIs and reduce their associated negative clinical consequences.

This study aimed to evaluate the prescriptions of inpatients with malaria for pDDIs prevalence, and their levels. Investigate the risk factors contributing towards pDDIs prevalence, and clinical relevance of pDDIs. Secondary aim was to identify monitoring parameters and develop management guidelines for the most frequent pDDIs.

## Methods

### Study settings and design

A retrospective cohort study was conducted at two tertiary care hospitals of Peshawar, Khyber Pakhtunkhwa, Pakistan such as Khyber Teaching Hospital (KTH) and Hayatabad Medical Complex (HMC). In healthcare system of Pakistan, tertiary care hospitals are more developed as compared to secondary or primary care hospitals. KTH and HMC are among the three major tertiary care hospitals where majority of the Khyber Pakhtunkhwa population visits for healthcare services. Malaria patients are more frequently observed in these two hospitals [4]. Malaria has been reported in high frequency in Peshawar in comparison to other cities of Khyber Pakhtunkhwa [22]. *Plasmodium vivax* and *Plasmodium falciparum* malaria are the common malaria forms found in these settings [4, 22]. Additionally, computerized drug interaction screening programmes and clinical pharmacy services are lacking in both the hospitals. Patient’s profiles are developed in hand written format and records are maintained manually.

### Patient selection criteria

Following were the inclusion criteria:

- Patients diagnosed with malaria and hospitalized during 2-year period (from 01 January 2015 to 31 December 2016).
- Patients aged ≥ 18 years.
- Both male or female patients.
- All medications, that were prescribed during hospitalization of the patient were included in analysis.

A total of 409 malaria patients were hospitalized during study period. Eleven patients’ profiles lacking relevant data (hospital admissions, patients’ demographics, diagnoses, comorbidities/complications, medications therapy, sign/symptoms, daily progress reports, and laboratory test reports) required for the study were excluded.

### Sample size calculation and sampling technique

Sample size was calculated by the following formula [23]:

$${\text{n }} = {\text{ Z}}^{ 2} {\text{P}}\left( { 1- {\text{P}}} \right)/{\text{d}}^{ 2}$$

Based on the above formula taking 52.8% [21] of anticipated prevalence, 95% confidence level, and 5% margin of error, a sample size of 383 was obtained. Whereas, a total of 398 patients were eligible for inclusion in the study during the study period. Non-probability consecutive sampling technique was used for collecting data.

### Data source

The following data were collected from the patients’ profiles such as hospital admissions, patients’ demographics, diagnoses, comorbidities/complications, medications therapy, sign/symptoms, and laboratory test reports.

### Medications profiles screening for pDDIs

Medicines prescribed to patients were evaluated for pDDIs using Micromedex Drug-Reax^®^ [24]. This software classifies drug interactions on the basis of severity- (contraindicated, major, moderate, and minor) and documentation-levels (excellent, good, and fair) [24]. Overall-prevalence of pDDIs as well as prevalence of pDDIs based on severity-levels were reported. Prevalence of pDDIs were explored by screening drug pairs per prescription.

### Clinical relevance

The clinical relevance of ten most frequent pDDIs was reported, by correlating potential adverse consequences of pDDIs with patients’ signs, symptoms and laboratory test results. The clinical manifestations were stratified based on dose differences of the interacting drugs. The following cut off points were used for defining higher daily doses, calcium containing products: ≥ 600 mg/3 L; ceftriaxone: ≥ 3 g; isoniazid: ≥ 300 mg; rifampin: ≥ 450 mg; pyrazinamide: ≥ 1500 mg; acetaminophen: ≥ 1 g; prochlorperazine: ≥ 15 mg; quinine: ≥ 1350 mg; ranitidine: ≥ 150 mg; metronidazole: ≥ 1500 mg; domperidone: ≥ 30 mg; dexamethasone: ≥ 24 mg; and ciprofloxacin: ≥ 800 mg. Potential adverse effects in this study were defined based on Medscape laboratory reference ranges and Wiley standard laboratory values, which are as follow: leukocytosis: total leukocyte count > 11,000/μL; elevated blood urea nitrogen (BUN): BUN ≤ 20 mg/dL; elevated serum creatinine: serum creatinine > 1.06 mg/dL; elevated alkaline phosphatase: > 126 U/L; elevated alanine aminotransferase: > 59 U/L (male), > 36 U/L (female); tachycardia: heart rate > 100 beats/min; hypotension: systolic blood pressure (BP) < 80 mmHg and/or diastolic BP < 50 mmHg; hypokalaemia: serum potassium < 3.5 mmol/L. Management guidelines and monitoring parameters were developed for the most prevalent pDDIs. Widespread (most common) and clinically important pDDIs were enlisted along with their potential adverse consequences.

The causal association between the adverse outcomes and top-10 interacting drug combinations was evaluated through Drug Interaction Probability Scale (DIPS). It guides by using a series of 10 questions to calculate a probability score. According to DIPS, the DDIs induced adverse outcomes are categorized as highly probable (> 8 score), probable (5–8 score), possible (2–4 score), or doubtful (< 2 score) [25, 26].

### Statistical analysis

Data were presented in the form of frequencies and percentages alone or with median and interquartile range (IQR), where appropriate. A statistical method of logistic regression analysis was used to calculate odds ratios (OR) for various risk factors of pDDIs such as patients’ gender, age, number of prescribed medicines, hospital stay, and comorbidities. Dependent variable in the model was exposure to pDDIs. While, patients’ characteristics (gender, age, number of prescribed medicines, hospital stay, and comorbidities) were taken as independent variables in the model. Odds ratios and 95% confidence intervals (CIs) were calculated for each independent variable. Univariate logistic regression analysis was run initially. Then, multivariate analyses were performed for variables with p-values of ≤ 0.15. A p-value of ≤ 0.05 was considered as statistically significant. SPSS-v23 was used for statistical analyses of the data.

## Results

### General characteristics of study patients

Patients’ demographics are presented in Table 1. Of 398 patients, males were more prevalent (51.8%). Most of the patients were aged 21–40 years (44.2%). A majority of patients was prescribed with ≥ 5 drugs (80.4%). Most frequent hospital stay was ≥ 4 days (64.6%). The median (IQR) age, prescribed drugs and hospital stay was 30 years (22–50), 7 drugs (5–9), and 4 days (3–6), respectively. Hypertension (n = 52), DM (45), urinary tract infections (34), hepatitis (23), and ischemic heart diseases (IHD) (15) were the most prevalent comorbidities of the studied patients (Table 1). Of 398 patients, 8.3% of the patients presented with falciparum malaria, 36.7% vivax malaria, while 55% were non-specific. While, 10.1% of the patients were presented with cerebral malaria one of the forms of severe/complicated malaria. Moreover, exposure to pDDIs stratified against the patient’s characteristics are also shown in Table 1. PDDIs prevalence was found similar in male and female patients. While, pDDIs were commonly reported in patients aged > 40 years, prescribed with ≥ 5 medicines, and hospitalization of > 5 days. Moreover, pDDIs were mostly reported in patients with DM and IHD as comorbidities.

**Table 1:** General characteristics of study subjects and exposure to potential drug–drug interactions

General characteristics	Patients: n (%)	Exposure to pDDIs [Patients: n (%)]
Gender		
Male	206 (51.8)	77 (37.4)
Female	192 (48.2)	71 (37)
Age (years)		
≤ 20	96 (24.1)	40 (41.7)
21–40	176 (44.2)	53 (30.1)
> 40	126 (31.7)	55 (43.7)
Median (interquartile range)	30 (22-50)	
Drugs prescribed		
< 5	78 (19.6)	5 (6.4)
≥ 5	320 (80.4)	143 (44.7)
Median (interquartile range)	7 (5–9)	
Hospital stay (days)		
≤ 3	141 (35.4)	32 (22.7)
4–5	144 (36.2)	56 (38.9)
> 5	113 (28.4)	60 (53.1)
Median (interquartile range)	4 (3–6)	
Number of comorbidities		
No comorbidities	179 (45)	–
1–2	187 (46.9)	–
≥ 3	32 (8)	–
Comorbidities		
Hypertension	52 (13.1)	20 (38.5)
Diabetes mellitus	45 (11.3)	27 (60)
Urinary tract infection	34 (8.5)	13 (38.2)
Hepatitis	23 (5.8)	11 (47.8)
Ischemic heart disease	15 (3.8)	9 (60)
Anaemia	13 (3.3)	3 (23.1)
Dengue fever	12 (3)	5 (41.7)
Meningitis	11 (2.8)	5 (41.7)
Respiratory tract infection	9 (2.3)	2 (22.2)
Thrombocytopenia	9 (2.3)	2 (22.2)
Typhoid	9 (2.3)	–
Bicytopenia	7 (1.8)	–
Acute gastroenteritis	6 (1.5)	–
Asthma	6 (1.5)	–
Tuberculosis	6 (1.5)	–
Acute kidney injury	5 (1.3)	–
Pancytopenia	5 (1.3)	–
Decompensated chronic liver disease	4 (1)	–
Pneumonia	4 (1)	–
Congestive cardiac failure	3 (0.8) each	–
Miscellaneous	72 (18)^a^	–

*pDDIs* potential drug–drug interactions

^a^In miscellaneous the following diagnosis were reported: chronic obstructive pulmonary disease, depression, encephalitis, epilepsy, goiter, hepatic encephalopathy, herpes labialis, post-natal endometriosis, thalassemia, deep vein thrombosis as n = 3 (0.8%) each. While, cholelithiasis, fits, nephropathy, pleural effusion as n = 2 (0.5%) each. However, achondroplasia, aortic stenosis, arthritis, atrial fibrillation, cellulitis, dementia, disseminated intravascular coagulation, down syndrome, endocarditis, eosinophilia, hyponatremia, hypothyroidism, immune thrombocytopenic purpura, leukemia, liver abscess, left ventricular failure, lymphoma, malignancy, menorrhagia, multiple myeloma, osteoporosis, post splenectomy, psychiatric disorder, rheumatic heart disease, renal tubular acidosis, systemic lupus erythematosus, spondylosis, sexually transmitted disease, stroke, thyrotoxicosis, tonsillitis, ulcerative colitis, urosepsis, Wilson disease as n = 1 (0.3%) each

### Prevalence of potential drug–drug interactions

**Fig. 1:**
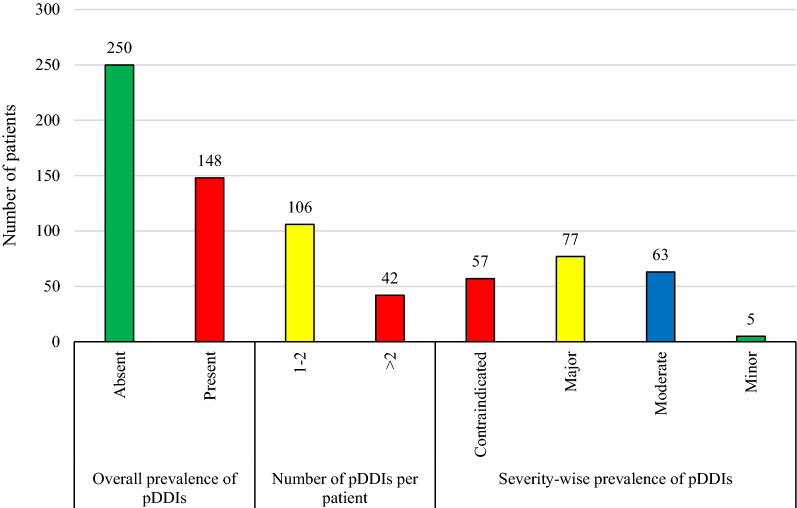
Prevalence of potential drug–drug interactions. pDDIs: potential drug-drug interactions. Data are presented in the form of frequencies. Overall-prevalence means the presence of at least one pDDI regardless of severity type. Study sample were 398 malaria patients. While, patients with pDDIs were 148 (overall prevalence of pDDIs = 37.2%). PDDIs prevalence was also reported based on severity-levels

Out of total 398 patients, 148 (37.2%) met at least one pDDI. Based on severity-wise prevalence, 19.3% patients were identified with at least one major-pDDI while, 15.8% with at least one moderate-pDDI. However, a smaller proportion of patients were found with contraindicated- (14.3%) and minor-pDDIs (1.3%) (Fig. 1).

### Levels of potential drug–drug interactions

**Fig. 2:**
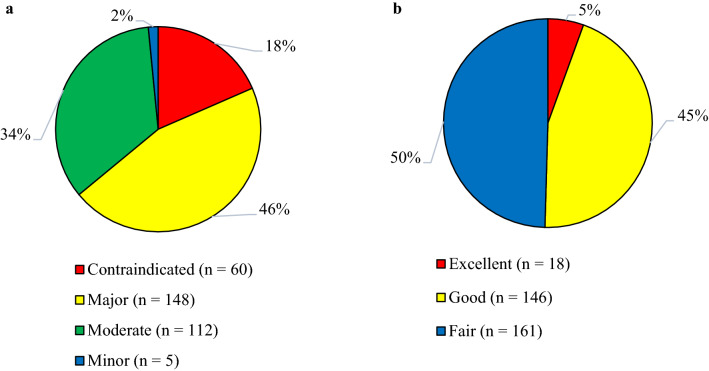
Levels of potential drug–drug interactions in patients with malaria. **a** Severity-levels of pDDIs. **b** Documentation-levels of pDDIs. pDDIs, potential drug–drug interactions. The total recorded pDDIs 325 were classified based on severity- and documentation-levels

Figure 2 illustrates categorization of pDDIs based on severity- and documentation-levels. Total number of interactions was 325, among which 45.5% were of major- and 34.5% moderate-severity. Based on documentation-levels, 49.5% were of fair and 44.9% good scientific-evidence.

### Risk factors of potential drug–drug interactions

Table 2 shows logistic regression analysis based on exposure to pDDIs. In the univariate logistic regression analysis, association for pDDIs was statistically significant with 5–6 prescribed medicines (p = 0.005), > 6 prescribed medicines (p < 0.001), hospital stay of 4–5 days (p = 0.003), and > 5 days hospitalization (p < 0.001). Moreover, concerning comorbidities, association of pDDIs with DM (p = 0.001) and IHD (p = 0.07) was statistically significant. In the multivariate logistic regression analysis, the association remained significant with 5–6 prescribed medicines (p = 0.01), > 6 prescribed medicines (p < 0.001), > 5 days hospitalization (p = 0.03), and DM (p = 0.04).

**Table 2:** Logistic regression analysis based on exposure to potential drug–drug interactions

Variables	Univariate analysis		Multivariate analysis	
	OR (95% CI)	p-value	OR (95% CI)	p-value
Gender				
Female	Reference		–	
Male	1 (0.7–1.5)	0.9	–	–
Age (years)				
≤ 20	Reference		Reference	
21–40	0.6 (0.4–1)	0.05	0.6 (0.3–1.1)	0.1
> 40	1.1 (0.6–1.9)	0.8	0.6 (0.3–1.1)	0.1
Drugs prescribed				
≤ 4	Reference		Reference	
5–6	4.3 (1.5–11.8)	0.005	3.9 (1.4–10.8)	0.01
> 6	17.9 (6.9–45.9)	< 0.001	14.1 (5.4–37.3)	< 0.001
Hospital stay (days)				
≤ 3	Reference		Reference	
4–5	2.2 (1.3–3.6)	0.003	1.5 (0.8–2.6)	0.2
> 5	3.9 (2.2–6.6)	< 0.001	1.9 (1.1–3.5)	0.03
Comorbidities				
Hypertension	1.1 (0.6–1.9)	0.8	–	–
Diabetes mellitus	2.9 (1.5–5.4)	0.001	2.2 (1–4.8)	0.04
Urinary tract infection	1.1 (0.5–2.2)	0.9	–	–
Hepatitis	1.6 (0.7–3.7)	0.3	–	–
Ischemic heart disease	2.6 (0.9–7.6)	0.07	2.4 (0.7–8.5)	0.2
Anaemia	0.5 (0.1–1.8)	0.3	–	–
Dengue fever	1.2 (0.4–3.9)	0.7	–	–
Meningitis	1.4 (0.4–4.7)	0.6	–	–
Respiratory tract infection	0.5 (0.09–2.3)	0.4	–	–
Thrombocytopenia	0.5 (0.09–2.3)	0.4	–	–

*CI* confidence interval, *OR* odds ratio

### Clinical relevance of potential drug–drug interactions

Table 3 presents daily prescribed dosage of the ten most frequent interacting drug pairs. In this study, the term high and low doses were used relatively. It was observed that the drugs were prescribed in varying doses and administration frequencies. Interacting drugs were prescribed more frequently in low doses, whereas, higher doses of the drugs were prescribed less frequently. Most frequent pDDIs along with their frequencies, proportions, potential adverse consequences and severity- and documentation-levels are presented in Additional file 1: Table S1. Most of the top ten pDDIs were of major severity (n = 7). While Additional file 2: Table S2 and Additional file 3: Table S3 enlists most prevalent anti-microbial agents (AMAs) and drugs besides AMAs, respectively. Artesunate (n = 378), quinine (63), artemether (26), lumefantrine (23), primaquine (18), amodiaquine (11), and chloroquine (9) were the commonly prescribed anti-malarial agents to these study patients (Additional file 2: Table S2).

**Table 3:** Dose regimen of the prescribed interacting drugs

Interacting pair	Dose categories^a^	Daily prescribed dose regimen	Number of patients
Calcium containing products—Ceftriaxone	Low + low	200 mg/L OD + 2 g OD ATD	10
	Low + low	200 mg/L BD + 2 g OD ATD	9
	Low + low	200 mg/L BD + 1 g BD ATD	8
	Low + high	200 mg/L OD + 2 g BD ATD	6
	Low + high	200 mg/L BD + 2 g BD ATD	5
	High + high	200 mg/L TDS + 2 g BD ATD	3
	High + low	200 mg/L TDS + 2 g OD ATD	3
	Low + high	200 mg/L OD + 3 g OD ATD	2
	High + low	1 g OD +2 g OD ATD	2
	Low + high	200 mg/L BD + 3 g OD ATD	1
	Low + high	200 mg/L BD + 4 g OD ATD	1
	High + high	1 g OD +2 g BD ATD	1
	Low + low	200 mg/L OD + 1 g OD ATD	1
Isoniazid–rifampin	High + high	300 mg OD + 600 mg OD	6
	Low + high	225 mg OD + 450 mg OD	2
	Low + low	150 mg OD + 300 mg OD	2
Pyrazinamide–rifampin	High + high	1600 mg OD + 600 mg OD	6
	Low + high	1200 mg OD + 450 mg OD	2
	High + low	500 mg TDS + 300 mg OD	2
Isoniazid–acetaminophen	High + high	300 mg OD + 500 mg TDS	2
	Low + high	300 mg OD + 500 mg TDS	2
	High + high	300 mg OD + 1 g OD	2
	Low + low	150 mg OD + 300 mg OD	1
	Low + high	150 mg OD + 500 mg TDS	1
	High + high	300 mg OD + 500 mg QID	1
Prochlorperazine–quinine	High + high	5 mg TDS + 600 mg TDS	4
	Low + low	5 mg BD + 600 mg BD	2
	High + high	5 mg TDS + 450 mg TDS	1
	High + low	5 mg TDS + 300 mg TDS	1
Cefpodoxime–ranitidine	Low + low	100 mg BD + 50 mg BD	5
	Low + high	100 mg BD + 50 mg TDS	2
Metronidazole–quinine	High + high	500 mg TDS + 600 mg TDS	5
	Low + low	400 mg TDS + 600 mg BD	1
Domperidone–ranitidine	High + low	10 mg TDS + 50 mg BD	4
	Low + high	10 mg BD + 50 mg TDS	1
	High + high	10 mg TDS + 50 mg TDS	1
Dexamethasone–rifampin	High + high	8 mg TDS + 600 mg OD	3
	Low + high	8 mg BD + 600 mg OD	1
	Low + low	4 mg TDS + 450 mg OD	1
Ciprofloxacin–metronidazole	High + low	500 mg BD + 500 mg TDS	3
	High + low	400 mg BD + 500 mg TDS	1
	Low + low	250 mg BD + 500 mg TDS	1

*OD* once a day, *BD* twice a day, *QID* four times a day, *TDS* three times a day, *ATD* alternate day

^a^The terms low and high doses were used relatively. For defining higher daily doses the following cut off points were used, calcium containing products: ≥ 600 mg/3 L; ceftriaxone: ≥ 3 g; isoniazid: ≥ 300 mg; rifampin: ≥ 450 mg; pyrazinamide: ≥ 1500 mg; acetaminophen: ≥ 1 g; prochlorperazine: ≥ 15 mg; quinine: ≥ 1350 mg; ranitidine: ≥ 150 mg; metronidazole: ≥ 1500 mg; domperidone: ≥ 30 mg; dexamethasone: ≥ 24 mg; and ciprofloxacin: ≥ 800 mg

In Table 4, specific clinical features (signs, symptoms and/or laboratory findings) and management guidelines/monitoring parameters [24, 27] for ten most frequent pDDIs are reported. The clinical features were stratified based on dose differences of the interacting drug pairs. Signs, symptoms and abnormalities in laboratory findings indicating poor response and nephrotoxicity were detected in patients with the interaction, calcium containing products + ceftriaxone. Patients with the interactions pyrazinamide + rifampin, isoniazid + rifampin, and isoniazid + acetaminophen, were observed with the signs/symptoms of hepatotoxicity such as weight loss, anorexia, hepatomegaly, pale, weakness, body aches, and ascites, and abnormalities in laboratory tests, such as elevated alkaline phosphatase and elevated alanine aminotransferase. Patients with the interacting pair, prochlorperazine + quinine, metronidazole + quinine, domperidone + ranitidine, and ciprofloxacin + metronidazole, were observed with clinical features and abnormalities in laboratory tests suggesting QT interval prolongation. Clinical features suggesting poor response of the drugs were observed in patients with the interacting pairs cefpodoxime + ranitidine and dexamethasone + rifampin. Table 4 further enlists monitoring parameters and management guidelines specifically for each interacting pair. Adverse consequences for the most frequent pDDIs were nephrotoxicity, hepatotoxicity, QT interval prolongation, and decreased therapeutic response. In general, monitoring parameters for the associated adverse effects includes related signs/symptoms and abnormal laboratory findings such as liver function tests, ECG, and renal function tests. Most of these associated adverse consequences can be managed by discontinuing the combination or adjusting the dose.

**Table 4:** Clinical relevance and management guidelines/monitoring parameters of most frequent potential drug–drug interactions in patients with malaria

Interactions^a^	Dose categories^a^	Signs and symptoms^a^	Laboratory investigations^a^	Management guidelines/monitoring parameters
Calcium containing products—Ceftriaxone (52)	High + high (4)	Fever (3), sepsis (1)	Elevated BUN (1), elevated serum creatinine (1), leukocytosis (2)	Avoid mixing or administering ceftriaxone concomitantly with calcium-containing IV solutions or infusions in the same IV administration line through a Y-site. Monitor for signs of nephrotoxicity, thrombosis, precipitates deposition in lungs, or decreased ceftriaxone effectiveness
	High + low (5)	Fever (3)	Elevated BUN (3), leukocytosis (1)	
	Low + high (15)	Fever (4), cough (4), congested chest (2), chest pain (1), breathing difficulty (1)	Elevated BUN (5), elevated serum creatinine (5), leukocytosis (5)	
	Low + low (28)	Cough (6), fever (4), chest pain (3), orthopnea (2), tachypnea (1), wheezing (1)	Elevated BUN (5), elevated serum creatinine (7), leukocytosis (3)	
Isoniazid–rifampin (10)	High + high (6)	Vomiting (1), body aches (1), left hypochondrium pain (1)	Elevated ALT (1), elevated ALP (2)	Monitor for signs and symptoms of hepatotoxicity such as jaundice, vomiting, fever, and anorexia. Also monitor baseline and periodic LFTs
	Low + high (2)	Anaemia (1), pale (1), weakness (1), anorexia (1), body aches (1)	Elevated ALP (1)	
	Low + low (2)	Body aches (1), pale (1), weight loss (1), ascites (1), hepatomegaly (1), anorexia (1)	Elevated ALT (1), elevated ALP (2)	
Pyrazinamide–rifampin (10)	High + high (6)	Vomiting (1), body aches (1), left hypochondrium pain (1)	Elevated ALT (1), elevated ALP (2)	Monitor for signs and symptoms of hepatotoxicity such as jaundice, vomiting, fever, and anorexia. Also monitor baseline and periodic LFTs
	Low + high (2)	Anaemia (1), pale (1), weakness (1), anorexia (1), body aches (1)	Elevated ALP (1)	
	Low + low (2)	Body aches (1), pale (1), weight loss (1), ascites (1), hepatomegaly (1), anorexia (1)	Elevated ALT (1), elevated ALP (2)	
Isoniazid–acetaminophen (9)	High + high (5)	Vomiting (1), body aches (1), left hypochondrium pain (1)	Elevated ALT (1), elevated ALP (1)	Monitor for signs and symptoms of hepatotoxicity such as jaundice, vomiting, fever, and anorexia. Also monitor baseline and periodic LFTs. Avoid concomitant administration of hepatotoxic drugs
	Low + high (3)	Anorexia (2), pale (1), anaemia (1), vomiting (1), weakness (1), body aches (1), ascites (1), hepatomegaly (1)	Elevated ALT (1), elevated ALP (2)	
	Low + low (1)	Body aches (1), pale (1), weight loss (1)	Elevated ALP (1)	
Prochlorperazine–quinine (8)	High + high (5)	Tachycardia (4), hypotension (3), hypertension (1)	Hypokalemia (1)	Monitor ECG and signs and symptoms of QT interval prolongation, specifically in patients at higher risk. Concomitant administration of QT interval prolonging drugs needs to be avoided
	High + low (1)	Hypotension 1)		
	Low + low (2)	Hypotension (2), tachycardia (1), chest pain (1), confusion (1)	Hypokalemia (1)	
Cefpodoxime–ranitidine (7)	Low + high (2)	Fever (1)	–	Administer cefpodoxime at least 2 h before ranitidine, or administer cefpodoxime with food. Monitor for improvement in patient condition
	Low + low (5)	Fever (2), urosepsis (1)	Leukocytosis (3)	
Metronidazole–quinine (6)	High + high (5)	Tachycardia (3), hypotension (3), hypertension (1), confusion (1), chest pain (1)	Hypokalemia (2)	Monitor ECG and signs and symptoms of QT interval prolongation, specifically in patients at higher risk. Concomitant administration of QT interval prolonging drugs needs to be avoided
	Low + low (1)	Chest pain (1), tachycardia (1), hypotension (1)	–	
Domperidone–ranitidine (6)	High + high (1)	Hypotension (1)	–	Monitoring for signs and symptoms of domperidone toxicity is suggested. Start domperidone at low dose then titrate gradually with caution. Discontinue domperidone if patient experiences syncope, palpitations, dizziness, or seizure. Also monitor ECG and signs and symptoms of prolonged QT interval
	High + low (4)	Tachycardia (4), hypertension (3), headache (2), confusion (1), hypotension (1)	–	
	Low + high (1)	Tachycardia (1), hypotension (1)	–	
Dexamethasone–rifampin (5)	High + high (3)	Irritable (3), hypertension (2), hypotension (1), fatigue (1), nausea (1), vomiting (1)	Elevated FBS (2)	Monitor for signs and symptoms of adrenal insufficiency. Adjust dose of dexamethasone, if given combine
	Low + high (1)	Drowsiness (1), hypotension (1)	–	
	Low + low (1)	Vomiting (1), fever (1), hypotension (1)	–	
Ciprofloxacin—metronidazole (5)	High + low (4)	Hypotension (3), tachycardia (2), hypertension (1), orthopnea (1), chest pain (1)	–	Monitor ECG and signs and symptoms of QT interval prolongation, specifically in patients at higher risk. Concomitant administration of QT interval prolonging drugs needs to be avoided
	Low + low (1)	Dizziness (1), tachycardia (1)	Hypokalemia (1)	

*BUN* blood urea nitrogen, *ALT* alanine aminotransferase, *ALP* alkaline phosphatase, *LFTs* liver function tests, *FBS* fasting blood sugar

^a^Frequencies are given in parenthesis and calculated among patients with respective interaction

According to DIPS, pDDIs with a score of 5 (probable) were observed in the following drug combinations: calcium containing products—ceftriaxone (n = 28; 53.8%), isoniazid–rifampin (n = 4; 40%), pyrazinamide–rifampin (n = 6; 60%), isoniazid–acetaminophen (n = 6; 66%), prochlorperazine–quinine (n = 5; 62%), cefpodoxime–ranitidine (n = 3; 42.9%), metronidazole–quinine (n = 3; 50%), domperidone–ranitidine (n = 3; 50%), dexamethasone–rifampin (n = 1; 20%), and ciprofloxacin–metronidazole (n = 2; 40%). While, the following interacting pairs were observed with a score of 6 (probable): calcium containing products—ceftriaxone (n = 16; 30.8%), isoniazid–rifampin (n = 3; 30%), pyrazinamide–rifampin (n = 2; 20%), isoniazid–acetaminophen (n = 2; 22%), cefpodoxime–ranitidine (n = 2; 28.6%), metronidazole–quinine (n = 1; 16.7%), domperidone–ranitidine (n = 1; 16.7%), dexamethasone–rifampin (n = 3; 60%), and ciprofloxacin–metronidazole (n = 3; 60%).

## Discussion

DDIs remains one of the therapeutic challenges among inpatients [8]. Studies addressing pDDIs issues among hospitalized patients with malaria are lacking. The prevalence of pDDIs reported in the current research is higher (37.2%) in comparison to that among patients with acquired immune deficiency (33.5%) [28], liver cirrhosis (21.5%) [12], and hypertension (21.1%) [13]. Contrary, it is lower (37.2%) as compared to that among patients with hypertension (48%) [29], DM (52.2%) [14], and bone marrow transplant (60%) [15]. Furthermore, in current study, prevalence of major-pDDIs is higher (19.3%) as compared to that reported among patients with cancer (16%) [16]. Whereas, it is lower in comparison to that reported among patients with liver cirrhosis (21.4%) [12], hepatitis C (30–44%) [20], and stroke (61%) [17]. Similarly, the prevalence of contraindicated-pDDIs in patients with malaria is also lower (14.3%) in comparison to the prevalence reported among patients with hepatitis C (16.7%) [30]. This contradiction may be due to variable study population, drug prescribing patterns, study design, considering pDDIs types, and drug interaction screening software. Considering the findings of this study, malaria patients are more at risk to pDDIs. Further, 36.7% of the study patients were presented with vivax malaria and 8.3% falciparum malaria and 10.1% were diagnosed as cerebral malaria. These findings showed that patients of the current study were severely ill and DDIs can further deteriorate patients’ condition. Published literature has proposed some evidence based approaches to minimize, prevent or manage DDIs in hospital settings, such as screening medication profiles for pDDIs by using computerized screening programmes [31], engaging clinical pharmacists in assessing patients’ medication profiles for pDDIs [32–34], procedure for structured assessment of pDDIs [35], and checking pertinent laboratory findings for clinical relevance of interactions [8, 36].

Healthcare professionals can manage adverse outcomes related to interactions, by taking into considerations the levels of interactions. In this study, pDDIs of major and moderate types were commonly observed, while concerning documentation levels, pDDIs of fair and good types were more prevalent. These findings are inconsistent with the findings from other studies [12, 21, 37]. This situation is alarming as the findings of this study warrant about the exposure of malaria patients towards negative consequences of pDDIs. Therefore, identifying the type of interaction, by healthcare professional is crucial for managing pDDIs, minimizing the related risk, and designing prophylactic measures for prevention.

Hospitalized patients with malaria receive a variety of medications for the management of underlying disease, related complications, and/or comorbid illnesses [5–7]. The findings of this study support that provision of multiple therapy has been positively associated with pDDIs prevalence [16, 37–39]. Moreover, the statistically significant association of pDDIs with prolong hospitalization reported by the current study is in accordance with the published reports [21, 40]. Furthermore, this study observed a significant association of pDDIs with DM as comorbidity of malaria. The reason is that, in patients with DM, such drugs are prescribed, having higher risk of DDIs [41]. Furthermore, most commonly prescribed anti-malarials agents in our study patients were artesunate, quinine, artemether, lumefantrine, primaquine, amodiaquine, and chloroquine. While, quinine, artemether and lumefantrine were involved in most frequent pDDIs (Additional file 1: Table S1). Therefore, malarial patients to whom these drugs are prescribed must be screened for DDIs. In this regard, hospitalized malaria patients having any of the above-mentioned risk factors are at higher risk to pDDIs. Healthcare professionals should have knowledge regarding the factors contributing towards pDDIs prevalence. This will help in reducing the risk of pDDIs—patients more at risk to pDDIs should be individualized to improve drug therapy and reduce the adverse outcomes of pDDIs.

All types of pDDIs are not clinically significant. Hence, developing the list of clinically significant DDIs of the drugs used by patients with malaria is of immense need. The list will be helpful for the healthcare professionals for selective screening and identification of DDIs. Further, physician’s understanding and knowledge of DDIs helps in reducing the occurrence of associated adverse effects, providing quality care, adjusting therapeutic regimen, and avoiding related medicolegal concerns. Moreover, the frequently identified pDDIs may results in serious adverse outcomes such as hepatotoxicity, QT interval prolongation, hypoglycaemia, hyperglycaemia, bleeding, hypertension, reduction in therapeutic effectiveness, and drug’s toxicity. This is of particular concern because of associated risk of harm to patient.

A particular strength of this study is the assessment of clinical relevance of pDDIs. A limited number of studies focused on such an evaluation. Clinical relevance presents possible consequences of DDIs on clinical indicators/features and laboratory findings. In addition, clinical relevance also highlights the importance of screening medication list for DDIs—enlightened by published literature [32, 36, 39]. Assessing patients’ abnormal signs/symptoms and laboratory investigations help in monitoring the adverse consequences associated with DDIs. The potential negative consequences of ten most frequent pDDIs, observed in this study and published reports, emphasis the need of monitoring patients using these combinations [10, 42, 43]. In this study, doses of the interacting drugs have also been considered. Relatively higher doses of the interacting drugs may potentiate the harmful effects of the DDIs. This report showed that adverse effects were commonly observed among patients with higher doses of the interacting drugs. Adverse consequences related to DDIs can be reduced by checking patients’ clinical manifestations and laboratory reports. In this study, most of the pDDIs have a DIPS of 5 or 6, which means adverse effects were probably associated with the DDIs. DDIs with a high percentage of DIPS were more likely involved in clinically relevant interactions and adverse outcomes. Causality analysis of adverse events with DDIs will help in finding the cause of DDIs and managing the adverse effects. Thus, this aspect of therapy needs appropriate attention. Furthermore, monitoring parameters and/management guidelines for DDIs will be helpful for healthcare professionals to assess and manage DDIs in malaria patients. Additionally, this study can be extrapolated to other malarial patients hospitalized in Pakistani as well as other countries setup except those countries having variable malaria types and prescribing pattern [44, 45]. The diverse comorbidity profile [46], disease pattern [22, 47], and similar malaria type and prescribing pattern [46–48] will results in almost similar prevalence of pDDIs. A large amount of data was collected from two hospitals—which are major tertiary care hospitals of the Province receiving maximum number of patients from whole of the Province.

Potential limitations of this study include inclusion of inpatients. As in hospitals, patients with malaria are chiefly admitted for the treatment of related signs/symptoms/complications or various comorbid illnesses. The pDDIs identified in this study are primarily associated with the use of medications for the management of such issues. Therefore, the findings of this study may not be generalizable to ambulatory patients in whom the drug utilization, drug interaction, and disease pattern possibly are different. Moreover, in the current study, the term pDDIs has been used as; DDIs were not actually observed. If such assessment, is made prospectively it will have positive clinical outcomes. Data are scarce regarding adverse clinical outcomes produced by drug interactions. However, in published literature some retrospective studies are available highlighting the importance of such an evaluation [9, 49].

## Conclusions

PDDIs are commonly observed in patients with malaria due to prescription of drugs having higher risk of pDDIs. Healthcare professional’s knowledge about the most common pDDIs could help in preventing pDDIs and their associated negative effects. Pertinent clinical parameters, such as laboratory findings and signs/symptoms need to be checked, particularly in patients with polypharmacy, longer hospital stay, and diabetes mellitus. Careful monitoring for adverse outcomes as well as prescribing drugs with a low risk for pDDIs are significant measures to decrease adverse effects associated with DDIs.

## Supplementary information

**Additional file 1: Table S1.** Description of the most frequent potential drug–drug interactions in patients with malaria.

**Additional file 2: Table S2.** Most frequently prescribed antimicrobial agents among patient with malaria.

**Additional file 3: Table S3.** Most frequently prescribed drugs (other than antimicrobials) among patient with malaria.

## Data Availability

The datasets used and/or analysed during the current study are available from the corresponding author on reasonable request.

## Abbreviations

AMAs: Antimicrobial agents
ALP: Alkaline phosphatase
ATD: Alternate day
ALT: Alanine aminotransferase
BD: Twice a day
BP: Blood pressure
BUN: Blood urea nitrogen
CI: Confidence interval
DDIs: Drug–drug interactions
DM: Diabetes mellitus
FBS: Fasting blood sugar
IHD: Ischemic heart diseases
IQR: Interquartile range
LFTs: Liver function tests
OD: Once a day
OR: Odds ratios
pDDIs: Potential drug–drug interactions
QID: Four times a day
TDS: Three times a day
