# Supplementary material for: Potential drug–drug interactions associated with adverse clinical outcomes and abnormal laboratory findings in patients with malaria
Source: Malar J. 2020 Aug 31;19:316. doi: 10.1186/s12936-020-03392-5 (PMC7461345; doi:10.1186/s12936-020-03392-5)
Supplement: Supplementary file 1 — Additional file 1: Table S1. Description of the most frequent potential drug–drug interactions in patients with malaria. [file 12936_2020_3392_MOESM1_ESM.docx]

**Additional Table 1.** **Description of the most frequent potential drug–drug interactions in patients with malaria**

| **Interacting pair** | **n (%)** | **Severity** | **Documentation** | **Adverse outcomes** |
| --- | --- | --- | --- | --- |
| Calcium containing products – Ceftriaxone | 52 (33.1) | Contraindicated | Good | Formation of ceftriaxone-calcium precipitates and is contraindicated in neonates |
| Isoniazid – Rifampin | 10 (6.1) | Major | Good | Hepatotoxicity |
| Pyrazinamide – Rifampin | 10 (6.1) | Major | Good | Hepatotoxicity |
| Isoniazid – Acetaminophen | 9 (5.5) | Major | Excellent | Hepatotoxicity |
| Prochlorperazine – Quinine | 8 (4.9) | Major | Fair | QT-interval prolongation |
| Cefpodoxime – Ranitidine | 7 (4.3) | Moderate | Good | Decreased cefpodoxime effectiveness |
| Metronidazole – Quinine | 6 (3.7) | Major | Fair | QT-interval prolongation |
| Domperidone – Ranitidine | 6 (3.7) | Major | Fair | QT-interval prolongation |
| Dexamethasone – Rifampin | 5 (3.1) | Moderate | Good | Decreased dexamethasone effectiveness |
| Ciprofloxacin – Metronidazole | 5 (3.1) | Major | Fair | QT-interval prolongation |
| Insulin – Metformin | 5 (3.1) | Moderate | Fair | Hypoglycemia |
| Domperidone – Quinine | 5 (3.1) | Major | Fair | QT-interval prolongation |
| Dexamethasone – Diclofenac | 4 (2.5) | Major | Fair | Gastrointestinal ulcer or bleeding |
| Artemether/ Lumefantrine – Domperidone | 4 (2.5) | Major | Fair | QT-interval prolongation |
| Aspirin – Clopidogrel | 3 (1.8) | Major | Fair | Increased risk of bleeding |
| Acyclovir – Divalproex | 3 (1.8) | Moderate | Good | Decreased valproic acid plasma concentrations and potential increased seizure activity |
| Aspirin – Bisoprolol | 3 (1.8) | Moderate | Good | Increased blood pressure |
| Calcium – Cefpodoxime | 3 (1.8) | Moderate | Fair | Decreased cefpodoxime effectiveness |
| Alprazolam – Omeprazole | 3 (1.8) | Moderate | Fair | Benzodiazepine toxicity (central nervous system depression, ataxia, lethargy) |
| Bisoprolol – Insulin | 3 (1.8) | Moderate | Good | Hypoglycemia or hyperglycemia; decreased symptoms of hypoglycemia |
