# Supplementary material for: Potential drug–drug interactions associated with adverse clinical outcomes and abnormal laboratory findings in patients with malaria
Source: Malar J. 2020 Aug 31;19:316. doi: 10.1186/s12936-020-03392-5 (PMC7461345; doi:10.1186/s12936-020-03392-5)
Supplement: Supplementary file 2 — Additional file 2: Table S2. Most frequently prescribed antimicrobial agents among patient with malaria. [file 12936_2020_3392_MOESM2_ESM.docx]

Additional Table S2. Most frequently prescribed antimicrobial agents among patient with malaria

| **Class of drugs (ATC code)^a^** | **Drugs** | **Frequency** |
| --- | --- | --- |
| Antimalarial (P01B) | Artesunate | 378 |
|  | Quinine | 63 |
|  | Artemether | 26 |
|  | Lumefantrine | 23 |
|  | Primaquine | 18 |
|  | Amodiaquine | 11 |
|  | Chloroquine | 9 |
|  | Pyrimethamine | 1 |
| Second, third, and fourth generation cephalosporins (J01DC^b^, J01DD^c^, J01DE^d^) | Ceftriaxone | 241 |
|  | Cefoperazone | 45 |
|  | Cefpodoxime | 22 |
|  | Cefepime | 19 |
|  | Cefixime | 12 |
|  | Cefotaxime | 9 |
|  | Cefuroxime | 2 |
|  | Ceftazidime | 1 |
| Beta-lactam antibacterial, Penicillin (J01C) | Sulbactam | 45 |
|  | Amoxicillin | 13 |
|  | Piperacillin | 7 |
|  | Cloxacillin | 2 |
|  | Ampicillin | 2 |
|  | Benzylpenicillin | 1 |
| Drugs for treatment of tuberculosis (J04A) | Isoniazid | 11 |
|  | Rifampin | 10 |
|  | Pyrazinamide | 10 |
|  | Ethambutol | 10 |
|  | Streptomycin | 4 |
| Intestinal anti-infective (A07A) | Metronidazole | 29 |
|  | Vancomycin | 6 |
|  | Rifaximin | 6 |
|  | Miconazole | 2 |

-ATC, anatomical therapeutic chemical classification

-^a^ Drugs were grouped in accordance with the Anatomical Therapeutic Chemical Classification System.

-^b^ J01DC ATC code is for second generation cephalosporin.

-^c^ J01DD ATC code is for third generation cephalosporin.

-^d^ J01DE ATC code is for fourth generation cephalosporin.
