# Supplementary material for: Potential drug–drug interactions associated with adverse clinical outcomes and abnormal laboratory findings in patients with malaria
Source: Malar J. 2020 Aug 31;19:316. doi: 10.1186/s12936-020-03392-5 (PMC7461345; doi:10.1186/s12936-020-03392-5)
Supplement: Supplementary file 3 — Additional file 3: Table S3. Most frequently prescribed drugs (other than antimicrobials) among patient with malaria. [file 12936_2020_3392_MOESM3_ESM.docx]

Additional Table S3. Most frequently prescribed drugs (other than antimicrobials) among patient with malaria

| **Class of drugs (ATC code)^a^** | **Drugs** | **Frequency** |
| --- | --- | --- |
| Analgesic (N02) | Paracetamol | 328 |
|  | Tramadol | 25 |
|  | Aspirin | 13 |
| Electrolyte solution (B05XA) | Sodium chloride | 175 |
|  | Potassium chloride | 10 |
|  | Sodium bicarbonate | 4 |
|  | Calcium chloride | 4 |
| Proton pump inhibitor (A02BC) | Omeprazole | 109 |
|  | Esomeprazole | 62 |
|  | Pantoprazole | 2 |
|  | Rabeprazole | 1 |
| Vitamins (A11) | Multivitamin | 54 |
|  | Vitamin D | 17 |
|  | Pyridoxine | 8 |
|  | Methylcobalamin | 3 |
|  | Vitamin C | 2 |
|  | Vitamin B | 1 |
| H2-receptor antagonists (A02BA) | Ranitidine | 78 |
|  | Loratadine | 2 |
| Glucocorticoids (H02AB^b^, R03BA^c^) | Dexamethasone | 57 |
|  | Beclometasone | 8 |
|  | Prednisolone | 5 |
|  | Hydrocortisone | 3 |
|  | Fluticasone | 3 |
| Drugs used in diabetes (A10) | Insulin | 43 |
|  | Metformin | 13 |
|  | Gliclazide | 5 |
|  | Vildagliptin | 4 |
|  | Glimepiride | 3 |
|  | Sitagliptin | 1 |
|  | Pioglitazone | 1 |
| Anti-anemic preparations (B03) | Folic acid | 46 |
|  | Iron | 13 |
| Drugs for constipation (A06A) | Lactulose | 23 |
|  | Sodium picosulfate | 15 |
|  | Lactitol | 5 |
|  | Sodium biphosphate/sodium phosphate | 2 |
| Propulsive (A03FA) | Domperidone | 20 |
|  | Metoclopramide | 13 |
|  | Itopride | 7 |

-ATC, anatomical therapeutic chemical classification

-^a^ Drugs were grouped in accordance with the Anatomical Therapeutic Chemical Classification System.

-^b^ H02AB ATC code is for dexamethasone, prednisolone, and hydrocortisone.

-^c^ R03BA ATC code is for beclometasone and fluticasone.

- Dimenhydrinate (antiemetic) was among the most frequently prescribed drugs, but not found in ATC system.
